# Use artificial neural network to align biological ontologies

**DOI:** 10.1186/1471-2164-9-S2-S16

**Published:** 2008-09-16

**Authors:** Jingshan Huang, Jiangbo Dang, Michael N Huhns, W Jim Zheng

**Affiliations:** 1 Dept Biostatistics, Bioinformatics, and Epidemiology, Medical University of South Carolina, Charleston, SC 29425, USA; 2 Siemens Corporate Research, Princeton, NJ 08540, USA; 3 Dept Computer Science & Engineering, University of South Carolina, Columbia, SC 29208, USA

## Abstract

**Background:**

Being formal, declarative knowledge representation models, ontologies help to address the problem of imprecise terminologies in biological and biomedical research. However, ontologies constructed under the auspices of the Open Biomedical Ontologies (OBO) group have exhibited a great deal of variety, because different parties can design ontologies according to their own conceptual views of the world. It is therefore becoming critical to align ontologies from different parties. During automated/semi-automated alignment across biological ontologies, different semantic aspects, i.e., concept name, concept properties, and concept relationships, contribute in different degrees to alignment results. Therefore, a vector of weights must be assigned to these semantic aspects. It is not trivial to determine what those weights should be, and current methodologies depend a lot on human heuristics.

**Results:**

In this paper, we take an artificial neural network approach to learn and adjust these weights, and thereby support a new ontology alignment algorithm, customized for biological ontologies, with the purpose of avoiding some disadvantages in both rule-based and learning-based aligning algorithms. This approach has been evaluated by aligning two real-world biological ontologies, whose features include huge file size, very few instances, concept names in numerical strings, and others.

**Conclusion:**

The promising experiment results verify our proposed hypothesis, i.e., three weights for semantic aspects learned from a subset of concepts are representative of all concepts in the same ontology. Therefore, our method represents a large leap forward towards automating biological ontology alignment.

## Background

The fields of biological and biomedical research are characterized by great complexity and imprecise terminologies. To address this imprecision and to standardize descriptions of biological entities, extensive efforts have been dedicated toward ontology development. The most successful endeavor is the development of Gene Ontology (GO), a formal and structured language, by the GO Consortium [1]. GO has three independently structured, controlled vocabularies: *molecular functions *– activities, such as catalysis or binding, at the molecular level; *biological processes *– events accomplished by one or more ordered assemblies of molecular functions; and *cellular components *– components that are part of some larger objects, such as an anatomical structure or gene product group [2]. To coordinate GO and other ontology development for biomedical research, the Open Biomedical Ontologies (OBO) group has developed mechanisms to share different ontologies [3]. Many ontologies in OBO have been represented in both the OBO format and Web Ontology Language (OWL) as well.

Althoug being formal, declarative knowledge representation models, ontologies from OBO have exhibited great variety. This variety stems from the fact that different parties can design ontologies according to their own conceptual views of the world. Unless this heterogeneity problem is resolved, it will be very difficult, if not impossible, to relate different ontologies and take advantage of the integration thereafter [4,5]. Current efforts to integrate ontologies include: 1) *merging *– merge several ontologies into a single one; 2) *mapping *– relate similar concepts or relationships across different ontologies, resulting in a virtual integration; and 3) *alignment *– define relationships between terms in different ontologies. In fact, mapping is a speical kind of alignment, i.e., to define *equivalentClassOf *relationship between two ontologies. This paper concentrates on the challenge of finding equivalent concept pairs from different biological ontologies, which is one of the most significant tasks in biological ontology alignment.

According to the classification in [6], most schema alignment techniques can be divided into two categories: rule-based and learning-based. We briefly discuss these two categories by summarizing some well-known algorithms.

The rule-based schema alignment techniques consider schema information only, and different algorithms distinguish from each other in their specific rules. PROMPT [7] provides a semi-automatic approach to ontology merging. By performing some tasks automatically and guiding the user in performing other tasks, PROMPT helps in understanding and reusing ontologies. Dou et al. [8] view ontology translation as ontology merging and automated reasoning, which are in turn implemented through a set of axioms. The authors regard the ontology merging as taking the union of the terms and the axioms defining them, then adding bridging axioms through the terms in the merge. Cupid [9] discovers mappings between schema elements based on their names, data types, constraints, and schema structure. Cupid has a bias toward leaf structure where much of the schema content resides. The experimental results show a better performance than DIKE and MOMIS. Giunchiglia et al. [10] view match as an operator that takes two graph-like structures and produces a mapping between the nodes. They discover mappings by computing semantic relations, determined by analyzing the meaning which is codified in the elements and the structures. The hypothesis in [11] is that a multiplicity of ontology fragments can be related to each other without the use of a global ontology. Any pair of ontologies can be related indirectly through a semantic bridge consisting of many other previously unrelated ontologies. Huang et al. [12] extend this work to incorporate: extended use of WordNet; use of the Java WordNet Library API for performing run-time access to the dictionary; and reasoning rules based on the domain-independent relationships and each ontology concept's property list to infer new relationships.

The learning-based schema alignment techniques consider both schema information and instance data, and various kinds of machine learning techniques have been adopted. GLUE [13] employs machine learning techniques to find semantic mappings between ontologies. After obtaining the results from a Content Learner and a Name Learner, a Metalearner is used to combine the predictions from both learners. Then common knowledge and domain constraints are incorporated through a Relaxation Labeler, and the mappings are finally calculated. In addition, the authors extend GLUE to find complex mappings. Williams [14] introduces a methodology and algorithm, DOGGIE, for multiagent knowledge sharing and learning in a peer-to-peer setting. DOGGIE enables multiagent systems to assist groups of people in locating, translating, and sharing knowledge represented in ontologies. After locating similar concepts, agents can continue to translate concepts and then are able to share meanings. Soh [15] describes a framework for distributed ontology learning embedded in a multiagent environment. The objective is to improve communication and understanding among the agents while agent autonomy is still preserved. Agents are able to evolve independently their own ontological knowledge, and maintain translation tables through learning to help sustain the collaborative effort. Wiesman and Roos [16] present an ontology matching approach based on probability theory by exchanging instances of concepts. During each step of the matching process, the likelihood that a decision is correct is taken into account. No domain knowledge is required, and the ontology structure plays no role. Madhavan et al. [17] show how a corpus of schemas and mappings can be used to augment the evidence about the schemas being matched. Such a corpus typically contains multiple schemas that model similar concepts and their properties. They first increase the evidence about each element being matched by including evidence from similar elements in the corpus. Then they learn statistics about elements and their relationships to infer constraints.

Both rule-based and learning-based algorithms have disadvantages. The former ignore the information obtained from instance data. A more severe problem is the way this technique treats different semantic aspects. In general, ontologies are characterized by the aspects of concept name, concept properties, and concept relationships. These aspects have different contributions to understanding ontologies' semantics. Take BiologicalProcess ontology [18] for example: there is a rich set of *super*/*subClassOf *relationships (over 20,000); however, at the same time, numerical strings, which are hardly meaningful to machines, are adopted as concept names, "GO_0030838" for example. Therefore, it is essential to assign different weights to different semantic aspects if a more accurate and meaningful alignment result is favored. Unfortunately, current research has made use of human intervention and/or prior domain knowledge to define these weights.

The main problems for learning-based algorithms include a relatively longer running time (due to the learning phase), and the difficulty in getting enough and/or good-quality data. The knowledge bases, i.e., ontologies and/or databases, in biological and biomedical area are usually huge. For example, there are more than 126,000 concepts in NCI Thesaurus ontology [19]. The extremely large file size imposes a higher requirement on any alignment algorithm's efficiency. Moreover, most biological ontologies have very few instance data, if any at all. Even for those with instances, these instances are most likely to be in semi-structured or unstructured formats, and therefore difficult to use.

In this paper, we present a new approach to align biological ontologies that combines both rule-based and learning-based algorithms. Our contributions are in the following. (1) Our approach integrates an artificial neural network (ANN) technique in our algorithm, such that the weights mentioned above can be learned instead of being specified by a human in advance. (2) Moreover, our learning technique is carried out based on the ontology schema information alone, which distinguishes it from most other learning-based algorithms.

The rest of this paper is organized as follows. Section 2 gives an overview of our method, and discusses the challenges in applying machine learning techniques without instance data information; it also presents the details of our algorithm. Section 3 reports the experiments conducted and analyzes the results. Section 4 concludes with an outline of future work in aligning biological ontologies.

## Results and discussion

### Purpose of our experiments

Our hypothesis is, three weights for semantic aspects learned from a subset of concepts are representative of all concepts in the same ontology. In order to verify this, we need to show by our experiments: (1) the learning process itself is a correct one, i.e., three weights converge to certain values; and (2) the learned weights are meaningful, i.e., the resultant equivalent concept pairs have satisfactory performance on *Precision *and/or *Recall*.

### Test ontologies

Two real-world biological ontologies, BiologicalProcess and Pathway, are adopted as the test ontologies. They have 13922 and 571 concepts, respectively; in addition, most relationships are *super*/*subClassOf *ones.

### Experiment design and results

**Figure 1:**
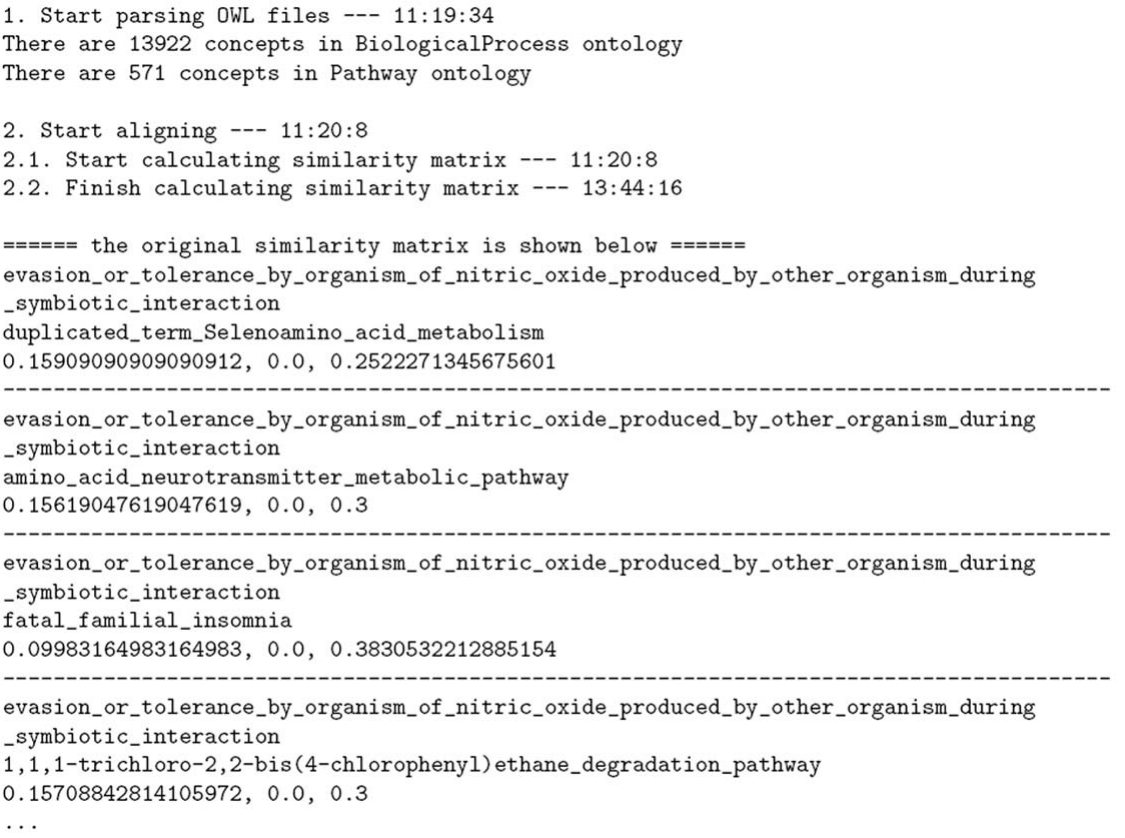
Program running log which contains portion of initial similarity matrix. Inside the matrix, for each concept pair, the first line is the concept from BiologicalProcess; the second line is the concept from Pathway; and the third line is their similarity values in concept name, concept properties, and concept relationships.

1. Both ontologies adopt numerical strings, "PW0000015" for example, as concept names, while the meaningful terms, "alzheimer_disease_pathway" for example, are embodied as labels. The purpose of this design is to avoid any potentially repeated concept names. We preprocessed both OWL files by replacing numerical strings with corresponding labels. Fortunately, there are no redundant concept names in either ontology.

2. An initial similarity matrix between these two ontologies was calculated. Some of this initial matrix is shown in Figure 1. There are 7,949,462 (13922 times 571) pairs in total. For each such pair, the first line is the concept from BiologicalProcess; the second line is the concept from Pathway; and the third line is their similarity values in concept name, concept properties, and concept relationships. For example, the similarity values between concepts "evasion_or_tolerance_by_organism_of_nitric_oxide_produced_by_other_organism_during _symbiotic_interaction" and "duplicated_term_Selenoamino_acid_metabolism" are: 0.15909090909090912, 0.0, and 0.2522271345675601.

3. We then randomly set *w*_1_, *w*_2_, and *w*_3_, and the learning rate *η *was also set to some certain value (see next section for detailed information of our settings). Finally, OAANN was provided with 30 pairs of equivalent concepts as training examples by domain experts (these training examples are listed in Table 1).

**Table 1:** Training examples in OAANN.

**Concepts from BiologicalProcess**		**Concepts from Pathway**
lipoprotein_metabolic_process (0042157)	vs.	lipoprotein_metabolic_pathway (0000482)
inositol_phosphate_metabolic_process (0043647)	vs.	inositol_phosphate_metabolic_pathway (0000154)
glutathione_catabolic_process (0006751)	vs.	glutathione_metabolic_pathway (0000134)
brassinosteroid_biosynthetic_process (0016132)	vs.	brassinosteroid_biosynthetic_pathway (0000552)
gamma-aminobutyric_acid_catabolic_process (0009450)	vs.	gamma-aminobutyric_acid_metabolic_pathway (0000412)
chondroitin_sulfate_biosynthetic_process (0030206)	vs.	chondroitin_sulfate_biosynthetic_pathway (0000195)
generation_of_precursor_metabolites_and_energy (0010497)	vs.	energy_metabolic_pathway (248)
acetylcholine_catabolic_process (0006581)	vs.	acetylcholine_metabolic_pathway (0000408)
cell_cycle_checkpoint(0000075)	vs.	cell_cycle_checkpoint_pathway (0000094)
DNA_replication_checkpoint (0000076)	vs.	G2/M_DNA_replication_checkpoint_pathway (0000385)
purine_metabolic_process (0006143)	vs.	purine_metabolic_pathway (0000031)
dopamine_catabolic_process (0042420)	vs.	dopamine_metabolic_pathway (0000409)
epinephrine_catabolic_process (0042419)	vs.	epinephrine_metabolic_pathway (0000441)
leukotriene_metabolic_process (0006691)	vs.	leukotriene_metabolic_pathway (0000464)
norepinephrine_catabolic_process (0042422)	vs.	norepinephrine_metabolic_pathway (0000442)
ganglioside_biosynthetic_process (0001574)	vs.	ganglioside_biosynthetic_pathway (0000164)
glycine_catabolic_process (0006546)	vs.	glycine_metabolic_pathway (0000440)
glucose_homeostasis (0042593)	vs.	glucose_homeostasis_pathway (0000553)
aspartate_metabolic_process (0006531)	vs.	aspartate_metabolic_pathway (0000439)
arachidonic_acid_metabolic_process (0019369)	vs.	arachidonic_acid_metabolic_pathway (0000460)
histamine_catabolic_process (0001695)	vs.	histamine_metabolic_pathway (0000411)
alanine_metabolic_process (0006522)	vs.	alanine_metabolic_pathway (0000438)
glycogen_biosynthetic_process (0005978)	vs.	glycogen_biosynthetic_pathway (0000532)
germ_cell_programmed_cell_death (0035234)	vs.	altered_programmed_cell_death (0000287)
C21-steroid_hormone_catabolic_process (0008208)	vs.	C21-Steroid_hormone_metabolic_pathway (0000070)
glycerophospholipid_metabolic_process (0006650)	vs.	glycerophospholipid_metabolic_pathway (0000354)
regulated_secretory_pathway (0045055)	vs.	regulated_secretory_pathway (0000537)
linoleic_acid_metabolic_process (0043651)	vs.	linoleic_acid_metabolic_pathway (0000523)
serotonin_catabolic_process (0042429)	vs.	serotonin_metabolic_pathway (0000410)
globoside_metabolic_process (0001575)	vs.	globoside_metabolic_pathway (0000196)

**Figure 2:**
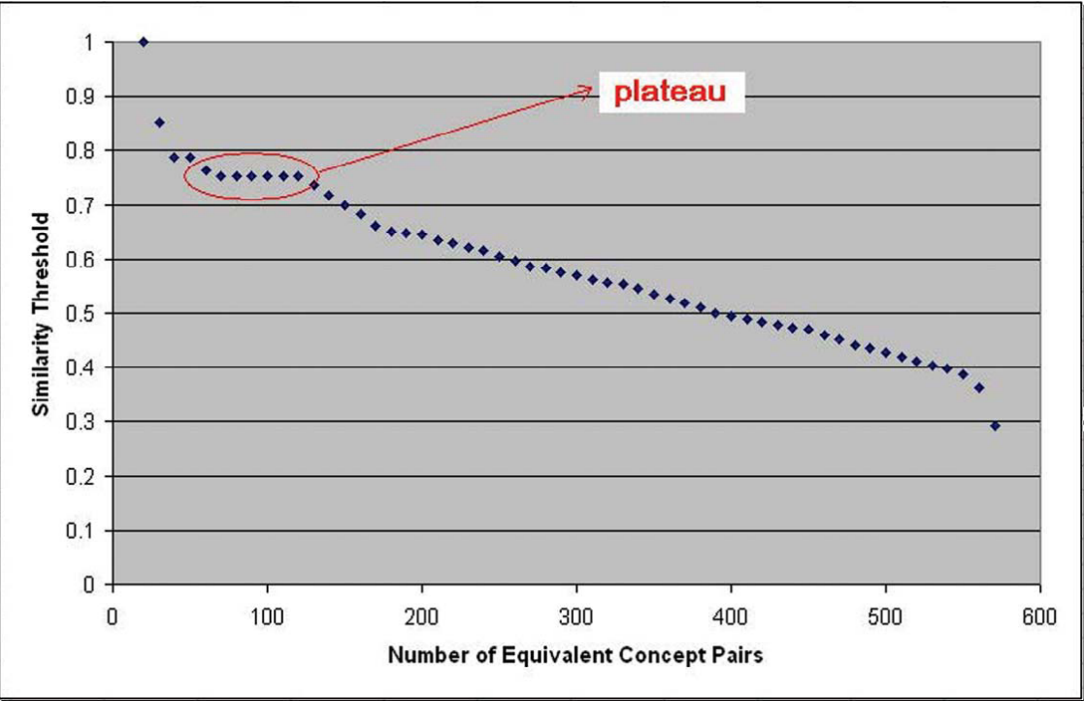
Plot of similarity threshold and equivalent concept pair number. The verizontal-axis value (0.75) at the beginning of the plateau in this figure was adopted as the similarity threshold. The intuition is: there is an initial drop followed by a plateau, which is in turn followed by a second drop. It is reasonable to conclude that threshold can possibly be assigned the value corresponding to the beginning of the plateau. Please refer to "Experiment Design and Results" section for more detailed explanation.

**Figure 3:**
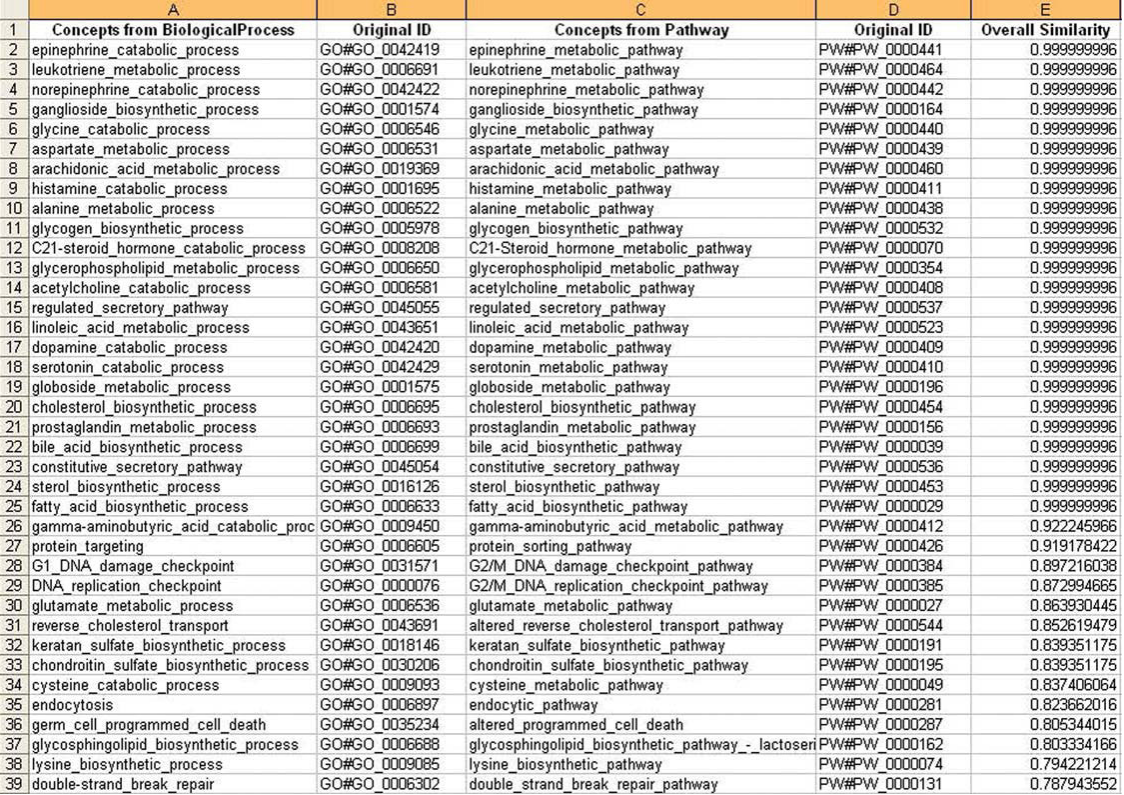
Portion of equivalent concept pairs output from OAANN. The final result, equivalent concept pairs between two test ontologies, was presented to domain experts, and Precision and Recall measurements were evaluated. Portion of these equivalent concept pairs are shown in this figure.

4. After a certain number of iterations (again, see next section for detailed information) in our ANN, **all three weights for semantic aspects converged**, and their values are 0.64, 0.01, and 0.35, respectively. These learned weights were then applied to recalculate the similarity matrix.

5. Out of the updated similarity matrix, a set of different thresholds for the similarity were chosen, i.e., from 1.00 to 0.29. We then used these thresholds to calculate equivalent concept pairs from the updated matrix.

6. A figure (Figure 2) was plotted out of the similarity threshold and the corresponding equivalent concept pair number. The verizontal-axis value (0.75) at the beginning of the plateau in this figure was adopted as the similarity threshold. We then output resultant equivalent concept pairs according to this threshold.

7. The final result of our methodology, equivalent concept pairs between two test ontologies, was presented to domain experts (same as those providing training examples), and *Precision *and *Recall *measurements were evaluated. Portion of these equivalent concept pairs are shown in Figure 3. Out of all 120 pairs, 108 pairs were agreed by domain experts, with another 19 pairs not from OAANN but suggested by domain experts. *Precision *and *Recall *are therefore 0.9 and 0.85, respectively.

### Analysis

**Figure 4:**
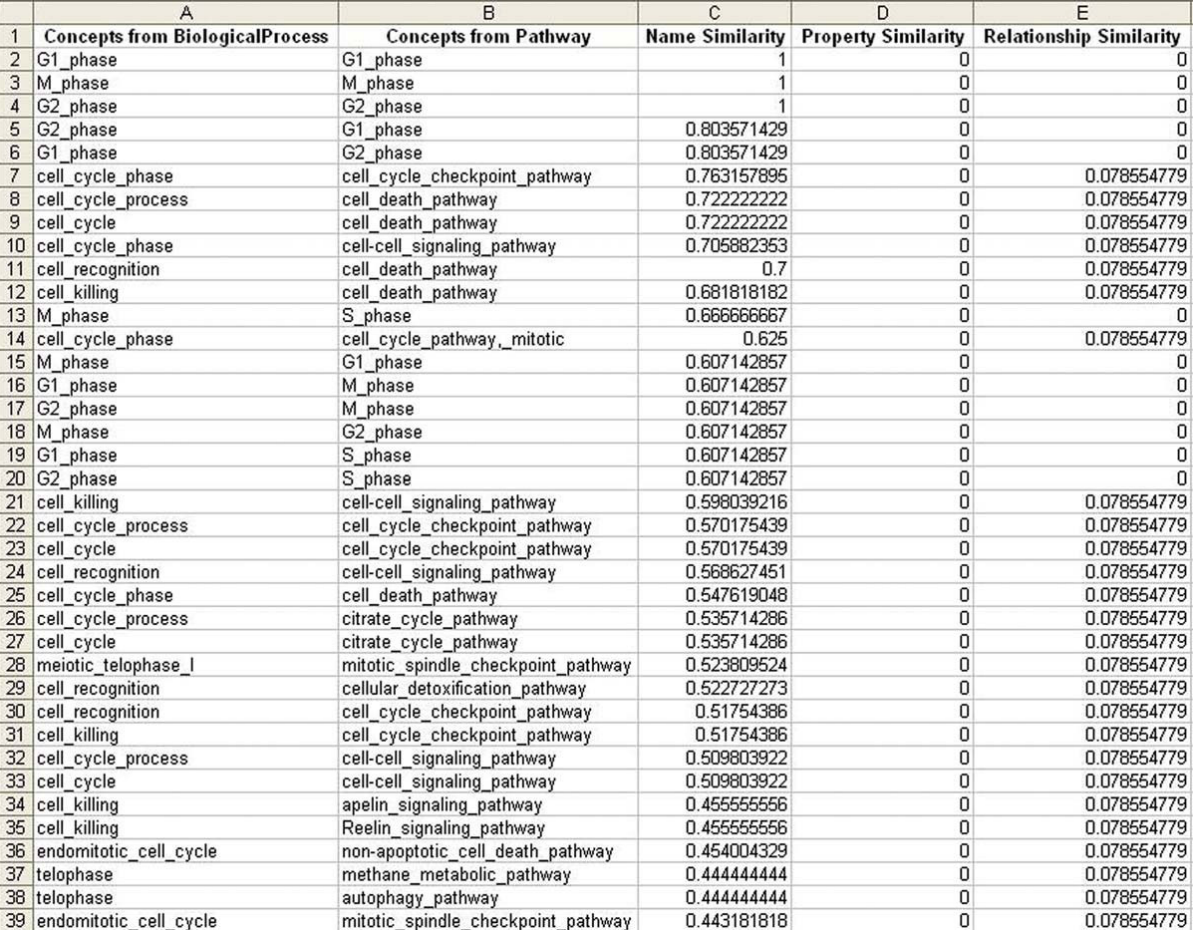
Similarity matrix sorted by concept name similarity. The similarity matrix was sorted by the "concept name similarity" column. Comparing this result with that in Figure 3 indicates that OAANN avoids the situation where string match alone is considered.

Based on the experiment results, it is clear that our hypothesis is validated since:

1. *w*_2 _has rather low value, reflecting the low contribution from concept properties. This exactly conforms to the characteristics of our test ontologies. Most properties in both test ontologies offer little help to the alignment process, because most property values are in semi-structured format and require natural language processing techniques before they can be used effectively.

2. We sorted the similarity matrix by the "concept name similarity" column, the result is shown in Figure 4. By comparing this result with that in Figure 3, it is obvious that our method avoids the situation where string match alone is considered. For example, "M_phase" and "S_phase" have a high similarity value in concept name, however, due to their low relationship similarity, 0 in this case, they are not considered as equivalent pair in the final output. On the other hand, "M_phase" and "M_phase" are in the equivalent pair output, although their overall similarity is lowered because their relationships are very different from each other – Pathway has a much more "flat" *super*/*subClassOf *hierarchy than BiologicalProcess. In a word, OAANN has a promising result by considering as many semantic aspects as possible, and by assigning appropriate weights for different aspects as well.

3. The percentage of training examples is around 24% (30 divided by 127). This small percentage reflects the feasibility and possibility of our method.

4. The adopted ANN structure in OAANN is not complicated because only three semantic aspects are considered. However, this structure is justified via experiment results. If we also consider other relationships in addition to the *super*/*subClassOf *ones, multilayer networks might be more appropriate, owing to their representational power.

5. We have tried different settings of initial weights, with a large range from (0.0, 0.0, 1.0) to (1.0, 0.0, 0.0). Our conclusion is, these weights converge to the same values, regardless of their initial values. In addition, varied learning rates will affect the speed of this convergence. The greater the learning rate, the smaller the number of iterations needed before the convergence. For example, if *η *is set to 0.05, 1000 iterations are needed; while if *η *is set to 0.1, only 700 iterations are necessary.

6. The curve shape in Figure 2 is: an initial drop followed by a plateau, which is in turn followed by a second drop. It is reasonable to conclude that threshold can possibly be assigned the value corresponding to the beginning of the plateau. The intuition is: the semantic similarity between non-equivalent concepts and that between equivalent concepts are different, and this difference could be remarkable enough to form a plateau.

7. OAANN adopts vectors to record semantic aspects, it is therefore not difficult to handle if more relationships are to be taken into consideration. What needs to be done is for us to expand the current vectors into more dimensions to hold more semantic aspects. Nevertheless, an ANN with multiple layers might be necessary in this case.

## Conclusion

Ontologies help in reconciling different views of independently developed and exposed data sources in biological and biomedical research area. Due to their inherent heterogeneity, ontologies need to be aligned before they can be integrated and used effectively. We present OAANN, a new alignment algorithm to overcome some disadvantages of both rule-based and learning-based approaches. Our contributions are: (1) we exploit an approach to learning the weights for different semantic aspects of ontologies, through applying an artificial neural network technique during the ontology alignment; and (2) we tackle the difficult problem of carrying out machine learning techniques without help from instance data. We explain and analyze our algorithm in detail, and our promising experiment results verify that OAANN represents a large leap forward towards automating biological ontology alignment.

Our focus has been on locating the equivalent concept pairs between two ontologies, leaving the other mapping tasks for future work, such as the discovery of parent-child concept pairs, the finding of sibling concept pairs, and so on. Another potential direction for the future work is to apply our approach in other biological ontologies, anatomy ontologies for example, where the bigger file size imposes some challenges with regard to OAANN's efficiency.

## Methods

### Overview of our approach

In our opinion, the semantics of an ontology concept are determined by three aspects: (1) the name of the concept; (2) the properties of the concept; and (3) the relationships of the concept. These three features together specify a conceptual model for each concept from the viewpoint of an ontology designer. For example, in the Pathway ontology [20], a concept has "altered_metabolic_pathway" as its name, two properties ("comment" and "label"), and seven relationships (*subClassOf *concept "classic_metabolic_pathway," *superClassOf *concepts "altered_amino_acid_metabolic_pathway," "altered_carbohydrate_metabolic_pathway," "altered_glycan_metabolic_pathway," "altered_lipid_metabolic_pathway," "altered_metabolic_pathway_of_cofactors_and_vitamins," and "altered_metabolic_pathway_of_other_amino_acids").

#### Challenges with existing alignment algorithms

Rule-based algorithms usually have the advantage of relatively fast running speed, but share the disadvantage of ignoring the additional information from instance data. In addition, there is a more serious concern for this type of algorithms. In order to obtain a helpful matching result from automated/semi-automated tools, more than one of the three semantic aspects mentioned above should be considered. If only one aspect is taken into account, then a meaningful matching result is unlikely to be acquired. Once two or more aspects are considered, it is unavoidable that corresponding weights for different aspects must be determined to reflect their different importance (or contributions) in ontology alignment. To the best of our knowledge, most existing rule-based algorithms make use of human heuristics and/or domain knowledge to predefine these weights. Moreover, once weights are determined, they are unlikely to be updated, or at most by trial-and-error.

While taking advantages of extra clues contained in instance data, the learning-based algorithms are likely to be slower. In addition, the difficulty in getting enough and/or good-quality data is also a potential problem. For example, in both BiologicalProcess and Pathway ontologies, there are barely instance data. On the other hand, it is very challenging for machines to learn to reconcile ontology structures, if machines are provided with schema information alone. The most critical challenge is that, because ontologies reflect their designers' conceptual views of part of the world, they exhibit a great deal of diversity. Identical terms can be used to describe different concepts, or vice versa, different terms can be assigned to the same concept. A more complicated situation is, even if the same set of terms are adopted, which is almost impossible in the real life, different designers can still create different relationships for the same concept, corresponding to their different conceptual views for this concept. Compared with schemas, instance data usually have a lot less variety.

#### Our solution

Based on the insight of the pros and cons of these two approaches, we present a new alignment algorithm, Ontology Alignment by Artificial Neural Network (OAANN), which combines rule-based and learning-based solutions. We integrate machine learning techniques, such that the weights of a concept's semantic aspects can be learned from training examples, instead of being ad-hoc predefined ones. In addition, in order to avoid the problem of missing instance data (either in quality or in quantity), which is common for real-world ontologies, our weight learning technique is carried out at the schema level instead of the instance level.

Our main idea is, given a pair of ontologies to be aligned, although it is true that a lot design diversity might exist, it is still reasonable to assume that the contributions of different semantic aspects to ontology understanding would hold across and therefore be independent of specific concepts. In fact, different contributions, which are the foundation for different weights, are characteristics of ontologies viewed as a whole. That is, during ontology alignment, weights are determined by ontologies, rather than by individual concepts. Therefore, we propose the following hypothesis: it is possible to learn these weights for *all* concepts by training examples from a *subset *of concepts.

Ontology alignment consists of many mapping tasks, for example, the discovery of parent-child concept pairs, the finding of sibling concept pairs, etc. OAANN concentrates on finding pairs of equivalent concepts as the first step. In addition, after the successful discovery of equivalent concept pairs, it is not difficult to design an algorithm to merge corresponding ontologies.

There are many kinds of relationships in ontologies, both domain-dependent and domain-independent, e.g., *superClassOf*, *subClassOf*, *partOf*, *contains*, etc. In this paper, we consider only the *super*/*subClassOf *relationships, which are the most common ones in most real-world ontologies. We plan to extend OAANN to include other relationships later. Due to the scalability of our approach (will be discussed later in this paper), this extension is relatively easy.

### Details of OAANN

We build a 3-dimension vector for each concept, and each dimension records one semantic aspect, i.e., concept name, concept properties, and concept relationships. When we match two concepts, we compare their contents in these three dimensions, and acquire the corresponding similarity in each dimension. Recall that our goal is to find the equivalent concept pairs.

#### Similarity in concept name

The similarity *s*_1 _between a pair of concept names is a real value in the range of [0, 1]. Some pre-processing on these two strings is performed before the calculation of *s*_1_. For example, the removal of hyphens and underscores.

If two names have an exact string matching, then *s*_1 _has a value of 1. Otherwise, *s*_1 _is calculated according to

(1) $$s_{1}=1-\frac{d}{l},$$

where *d *stands for the edit distance between two strings, and *l *for the length of the longer string.

#### Similarity in concept properties

Given two lists of concept properties (including those inherited from ancestors), *p*_1 _and *p*_2_, their similarity *s*_2 _is a real value in the range of [0, 1], and *s*_2 _is calculated according to

(2) $$s_{2}=\frac{n}{m},$$

where *n *is the number of pairs of properties matched, and *m *is the smaller cardinality of lists *p*_1 _and *p*_2_.

In order for a pair of properties (one from *p*_1 _and the other from *p*_2_) to be matched, their data types should be the same or compatible with each other (*float *and *double *for example), and the property names should have a similarity value greater than a threshold. Notice that here we use the same procedure as in Section to calculate the similarity between a pair of property names. In addition, we adopt the idea of "stable marriage" in determining the matched property pairs. That is, once two properties are considered matched, they both find the best matched one from the other property list. Imagine a similarity matrix built between *p*_1 _and *p*_2_; each time we pick up a pair with the maximum value in the matrix, say cell [*i*, *j*], and then discard row *i *and column *j*.

#### Similarity in concept relationships

We take into account only the *super*/*subClassOf *relationships. In order to obtain a better matching result, we make use of as much information as possible. For example, suppose there are two pairs of equivalent concepts, and the numbers of concepts in-between are different from each other, i.e., the ontology with more detailed design tends to have more intermediate concepts. If the direct parent alone is considered, the information from this multilayered parent-child hierarchy will be ignored. Therefore, we not only consider the direct parent of a concept, but also all ancestors (concepts along the path from a concept up to the root "Thing") of this concept as well. Descendants (direct and indirect children of a concept) are not taken into account, as that would lead to an infinite loop.

Given two lists of concept ancestors, *a*_1 _and *a*_2_, their similarity *s*_3 _is a real value in the range of [0, 1], and is obtained by first calculating the similarity values for pairwise concepts (one from *a*_1_, the other from *a*_2_, considering all combinations), then assigning the maximum value to *s*_3_. Notice that this is a recursive procedure but is guaranteed to terminate, because (1) the number of concepts is finite; and (2) we assume that "Thing" is a common root for the two ontologies being aligned.

#### Concept similarity matrix

After *s*_1_, *s*_2_, and *s*_3 _between two concepts, *C*_1 _and *C*_2_, are calculated, the similarity value *s *between *C*_1 _and *C*_2 _is obtained as the weighted sum of *s*_1_, *s*_2_, and *s*_3_:

(3) $$s=\sum_{i=1}^{3}\left(w_{i}s_{i}\right),$$

where $\sum_{i=1}^{3}w_{i}=1$. Notice that *w*_*i *_are randomly initialized and will be adjusted through a learning process (see Sec. 4.5 below).

For two ontologies being matched, $O_{1}$ and $O_{2}$, we calculate the similarity values for pairwise concepts (one from $O_{1}$, the other from $O_{2}$, considering all combinations). Then we build a *n*_1 _× *n*_2 _matrix ℳ to record all values calculated, where *n*_*i *_is the number of concepts in $O_{i}$. The cell [*i*, *j*] in ℳ stores the similarity value between the *i*^*th *^concept in $O_{1}$ and the *j*^*th *^concept in $O_{2}$.

#### Weight learning through ANN

The main purpose of OAANN is to try to learn different weights for three semantic aspects during the ontology alignment process. We design our learning problem as follows.

• Task *T*: align two ontologies (in particular, find equivalent concept pairs)

• Performance measure *P*: *Precision *and *Recall *measurements with regard to manual matching

• Training experience *E*: a set of equivalent concept pairs by manual matching

• Target function *V*: a pair of concepts → ℜ

• Target function representation: $\hat{V}(b)=\sum_{i=1}^{3}\left(w_{i}s_{i}\right)$

We choose ANN as our learning technique, based on the following considerations.

• Instances are represented by attribute-value pairs

• The target function output is a real-valued one

• Fast evaluation of the learned target function is preferable

### Network design

**Figure 5:**
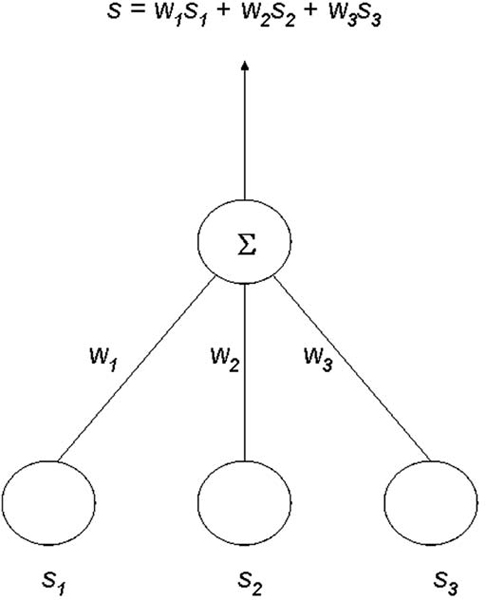
Neural network structure. The input into this network is a vector $\vec{s}$, which consists of *s*_1_, *s*_2_, and *s*_3_, representing the similarity in name, properties, and ancestors, respectively, for a given pair of concepts. *w*_*i *_are weights assigned to each input. The output from this network is *s*, the similarity value between these two concepts as given by Formula 3.

We adopt a two-layer 3 × 1 network in OAANN, as shown in Figure 5. The input into this network is a vector $\vec{s}$, which consists of *s*_1_, *s*_2_, and *s*_3_, representing the similarity in name, properties, and ancestors, respectively, for a given pair of concepts. The output from this network is s, the similarity value between these two concepts as given by Formula 3. Notice that a linear function might not be powerful enough to reflect the true relationships among *w*_*i*_. However, the delta rule converges toward a best-fit approximation to the target concept even when the training examples are not linearly separable [21]. If more relationships among ontology concepts are to be considered, then one or more layers of hidden units might need to be added to express a rich variety of nonlinear decision surfaces.

Initially, we obtain a concept similarity matrix ℳ for $O_{1}$ and $O_{2}$, with *w*_*i *_being initialized randomly. Then we randomly pick up a set of concepts from $O_{1}$, and find the corresponding equivalent concepts by a manual matching with $O_{2}$. Each of such manually matched pairs will be processed by OAANN, and the similarity values in name, properties, and ancestors for these two concepts are calculated and used as a training example to the network in Figure 5.

### Hypothesis space and our searching strategy

We regard the hypothesis space in this learning problem as a 3-dimensional space consisting of *w*_1_, *w*_2_, and *w*_3_, i.e., a set of weight vectors $\vec{w}$. Our objective is to find the weights that best fit the training examples. We adopt gradient descent (delta rule) as our training rule, and our searching strategy within the hypothesis space is to find the hypothesis, i.e., weight vector, that minimizes the training error with regard to all training examples. According to [21], a standard definition of the training error *E *of a hypothesis is given by

(4) $$E(\vec{w})\equiv \frac{1}{2}\sum_{d\in D}{\left(t_{d}-o_{d}\right)}^{2},$$

where *D *is the set of training examples, *t*_*d *_is the target output for training example *d*, and *o*_*d *_is the output of the network for *d*.

We customize the above formal definition according to the characteristics of our learning problem as follows. For any training example *d*, instead of a given target value *t*_*d*_, we need some other values. The intuition is that a given pair of manually matched concepts corresponds to a cell [*i*, *j*] in ℳ; therefore, the value of cell [*i*, *j*] should be the maximum one in both row *i *and column *j*. Suppose the maximum value for row *i *and column *j *are *t*_*r *_and *t*_*c*_, respectively. Then, our customized description of *E *is

(5) $$E(\vec{w})\equiv \frac{1}{2}\sum_{d\in D}{\left[(t_{r}-o_{d})+(t_{c}-o_{d})\right]}^{2}.$$

Accordingly, the weight update rule for gradient descent in OAANN is

(6) $$\Delta w_{i}\equiv \eta \sum_{d\in D}[(t_{r}-o_{d})+(t_{c}-o_{d})]s_{id},$$

where *η *is the learning rate, and *s*_*id *_is the *s*_*i *_value for a specific training example *d*.

#### Recalculate concept similarity and output equivalent concept pairs

After we obtain the learned weights, we apply them to recalculate the similarity matrix ℳ. We then pick up a threshold (see next section for details) and output the equivalent concept pairs according to this threshold. The resultant equivalent concept pairs between two ontologies are then presented to domain experts for verification.

## Competing interests

The authors declare that they have no competing interests.

## Authors' contributions

Jingshan Huang played a leading role in inventing the methodology, implementing the algorithm, analyzing the experiment results, and drafting the manuscript. Jiangbo Dang made significant contribution in inventing the methodology; he also contributed in analyzing the experiment results. Michael N. Huhns involved in the discussion of the methodology and algorithm. W. Jim Zheng and Jingshan Huang worked together to refine the methodology, apply it to biomedical ontologies, analyze the experimental results, and develop the manuscript.
